# In-hospital Routes of Acute Heart Failure Admissions During COVID-19

**DOI:** 10.3389/fcvm.2020.581458

**Published:** 2020-10-08

**Authors:** Gaetano Ruocco, Mauro Feola, Alberto Palazzuoli

**Affiliations:** ^1^Cardiology Division, Regina Montis Regalis Hospital, ASL-CN1, Cuneo, Italy; ^2^Cardiovascular Diseases Unit, Department of Internal Medicine, Le Scotte Hospital, University of Siena, Siena, Italy

**Keywords:** acute heart failure, ARDS, COVID-19, inhospital routes, emergency department

Coronavirus disease 2019 (COVID-19) is a new viral infection causing acute respiratory distress syndrome (ARDS) that has spread around the world counting 32,429,965 cases and 985,823 deaths as of September 26, 2020^1^. Because of the high percentage of COVID-19–related hospital admission, in Italy a reduction in cardiovascular disease hospitalization was observed, which could contribute to an increased rate of cardiovascular death out-of-hospital (1, 2). Acute heart failure (AHF) syndromes are characterized by a rapid symptom onset requiring fast hospitalization and treatment. Similarly, COVID-19–related ARDS needs hospital admission both for diagnosis and for treatment. However, dyspnea represents a common symptom of these pathological conditions, and for this reason, there is an unmet need of established in-hospital route to better manage the two diseases and to reduce in-hospital infection spread.

First, after presentation to the emergency department (ED), it is mandatory to distinguish two different routes: one is for known positive coronavirus patients and the other for unknown coronavirus patients. In case of positive coronavirus patients, they should recover in the “red zone” of the ED, where AHF patients should undergo clinical, laboratory, and instrumental assessment by clinicians with the support of a cardiologist advisor provided with showerproof single-use coat, gloves, facial protection, and FFP3 mask (3, 4). AHF diagnosis should be done following the latest guidelines criteria (5). Venous blood sample, arterial blood gas analysis, electrocardiogram, and chest x-ray are mandatory to identify the main diagnosis of each patient (viral infection ARDS or cardiovascular disease or cardiovascular involvement during COVID-19). Chest computed tomography scan should be performed according to clinical suspect of interstitial pneumonia. Monitoring electrocardiogram should be useful because of the high risk of arrhythmias in COVID-19 patients. In these patients, echocardiography should be performed in case of new-onset AHF (without medical history of HF), suspected pericardial tamponade, suspected acute pulmonary embolism, suspected AHF associated to acute coronary syndrome (ACS), and suspected acute valvular heart diseases. However, there are some limitations to using echocardiography in COVID-19 patients: (1) high risk of clinician contamination during echocardiography; (2) higher rate of echocardiography failure due to severe respiratory distress. The first concern should be avoided using the latest fast-echo protocol for COVID-19 patients with handheld tablet ultrasound, which provides appropriate information about cardiac conditions, limiting contact and contamination (6). The second concern should be overcome by the use of a contrast agent that enhances the identification/exclusion of ventricular thrombosis, abnormalities in wall motions, and computation of left ventricular ejection fraction (7). AHF treatment should be shared between the cardiologist advisor and ED clinicians, taking into account renal function deterioration, diuresis, electrolytes unbalance, and ARDS complication. ARDS complication should be managed in the “red zone” of the intensive care unit (ICU) together with ICU clinicians. In case of coronavirus-positive AHF patients, who do not show ARDS and do not require invasive ventilation (IV), these patients should be allocated into internal medicine ward, which should be organized as COVID-19 care unit. In this unit, patients should be managed by internal medicine clinicians together with a cardiologist advisor. In case of unknown for COVID-19 AHF patients, it is necessary to limit infection among patients and health care workers. It should be optimal to recognize a “gray zone” within the ED where patients should be screened for coronavirus. In this “gray zone,” patients should be far at least for 2 m, and healthcare workers should wear showerproof single-use coat, gloves, facial protection, and FFP3 mask (3, 4). Recovered patients should undergo nasopharyngeal swabbing at hospital admission and after 24–48 h. The cardiologist advisor should support clinicians for AHF diagnosis and treatment during coronavirus infection assessment, wearing showerproof single-use coat, gloves, facial protection, and FFP3 mask. In case of positive coronavirus nasopharyngeal swab, AHF patients should be managed in the “red zone” together with ED clinicians; these patients should be transferred to the ICU or COVID-19 care unit according to ARDS complications and the need for IV. In case of two negative coronavirus nasopharyngeal swab results, AHF patients could be transferred in the cardiology ward (“green zone”) and should be managed and treated by cardiologists. In the “green zone,” cardiologist and other healthcare workers should wear disposable paper gown, surgical masks, and gloves. All patients may wear surgical masks during the hospitalization period. Cardiac biomarkers (d-dimer, troponin, and natriuretic peptide) monitoring should be performed in all patients to recognize treatment efficacy, worsening heart failure, and acute cardiovascular complication related or not to COVID-19, such as acute pulmonary embolism or ACS (Figure 1).

**Figure 1:**
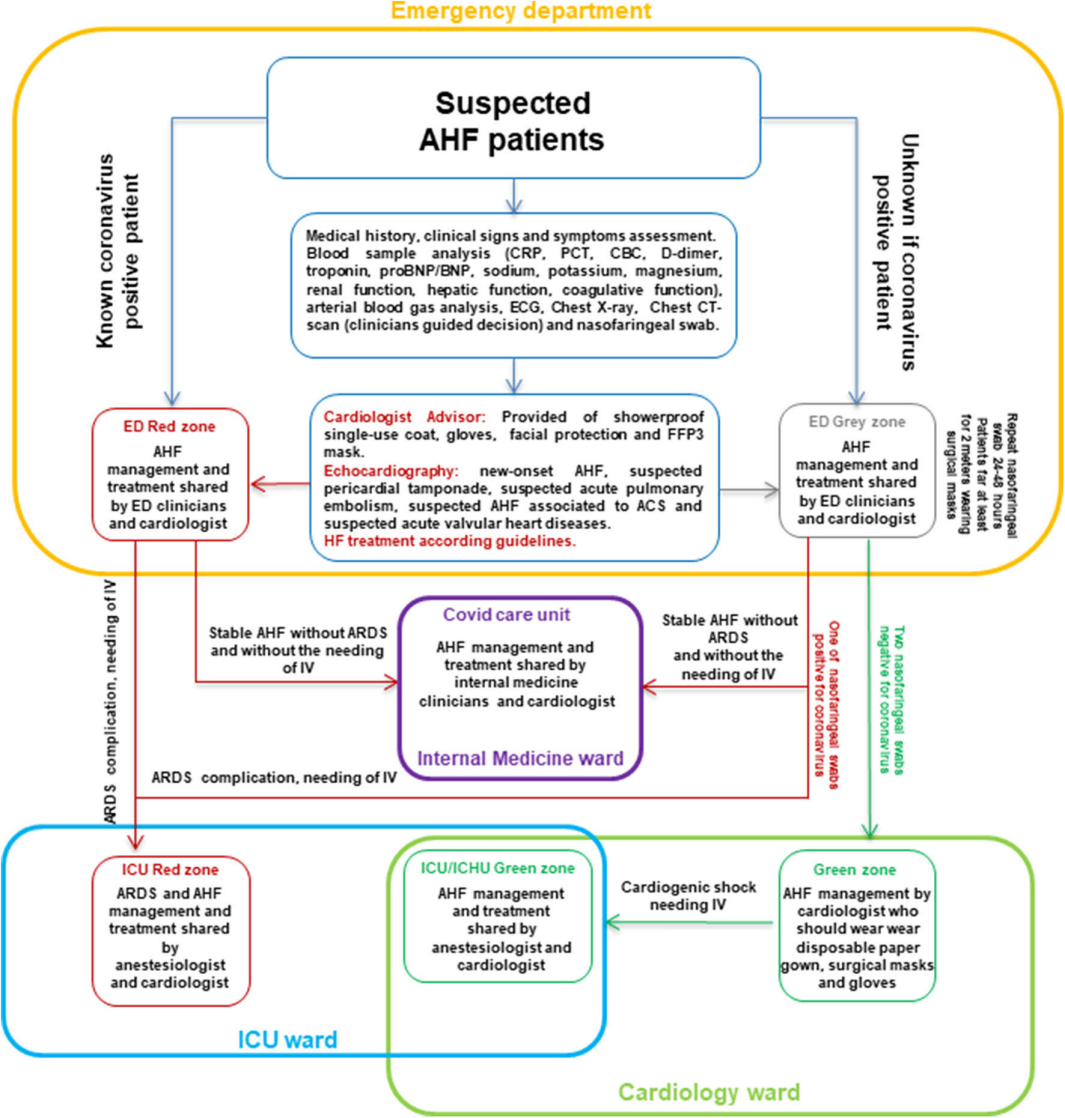
Flow chart for AHF patients hospital admission during COVID-19. ACS, acute coronary syndrome; AHF, acute heart failure; ARDS, acute respiratory distress syndrome; BNP, B-type natriuretic peptide; CBC, count blood cell; CRP, C-reactive protein; CT, computed tomography; ECG, electrocardiogram; ED, emergency department; ICU, intensive care unit; ICHU, intensive care heart unit; IV, invasive ventilation; PCT, procalcitonin.

## Author Contributions

GR give a substantial contribution in conception, design, writing, and revising the manuscript. MF and AP revising manuscript. All authors contributed to the article and approved the submitted version.

## Conflict of Interest

The authors declare that the research was conducted in the absence of any commercial or financial relationships that could be construed as a potential conflict of interest.

## Footnotes

^1^https://covid19.who.int/
